# The mediating role of social distance on the association between audience-protagonist similarity and fear: a case of fear of COVID-19

**DOI:** 10.3389/fpsyg.2026.1823941

**Published:** 2026-10-14

**Authors:** Inna Levy, Irene Razpurker-Apfeld, Lipaz Shamoa-Nir, Keren Cohen-Louck

**Affiliations:** 1 Department of Criminology, Ariel University, Ariel, Israel; 2 Department of Interdisciplinary Studies, Zefat Academic College, Zefat, Israel

**Keywords:** audience-protagonist similarity, construal level theory, fear of COVID-19, psychological distance, social distance

## Abstract

**Objective:**

This study examined the effects of news content on fear of COVID-19 by examining perceived social distance and audience-protagonist similarity.

**Method:**

Participants (*N* = 78) read a fictitious news story about a COVID-19 patient (similar/dissimilar protagonist) and then rated social distance regarding this protagonist and their fear of COVID-19.

**Results:**

The results showed that participants exposed to a similar protagonist reported significantly lower perceived social distance, and that social distance was moderately negatively correlated with fear of COVID-19. Social distance mediated the association between audience-protagonist similarity and fear of COVID-19.

**Conclusion:**

These findings provide insights into how exposure to health-related news content can impact fear of disease.

## Introduction

This study focuses on the effects of news content on fear of a viral pandemic. Viral pandemics are a reoccurring phenomenon, and human history includes many cases of viral and deadly pandemics (Bloom and Cadarette, 2019; Malta et al., 2020; Cohen-Louck and Levy, 2021), including 21st-century outbreaks such as avian flu, SARS, HIV, swine flu (H1N1), Ebola, and Zika (Moghadami, 2017; Tucci et al., 2017). Viral pandemics induce fears, anxiety, and stress (Shigemura et al., 2020), and their effects on public mental health can be more prolonged and significant than the physical effects of viral diseases (Reardon, 2015; Shigemura et al., 2020). Similarly, the manifestations and effects of the COVID-19 pandemic went beyond the adverse physical aspects of the virus (Cohen-Louck and Levy, 2021) and caused mass trauma (Gonçalves et al., 2020; Cohen-Louck, 2022), and high levels of fear of COVID-19 (Ho et al., 2020; Cohen-Louck and Levy, 2021; Levy and Cohen-Louck, 2021). It is essential to study the effects of news content on fear of COVID-19 because fear of COVID-19 is associated with individual mental health (Presti et al., 2020; Shapiro et al., 2020; Braun-Lewensohn et al., 2021; Levy and Cohen-Louck, 2021; Levy, 2022; Cohen-Louck and Levy, 2023) and individual commitment to use preventive measures (Muller, 2012; e.g., wearing a mask, hygiene practices; e.g., Heiat et al., 2021).

Furthermore, media outlets are vital in disseminating information during mass disasters (Cohen-Louck and Levy, 2021; Horesh and Brown, 2020) and promoting preventive measures during viral pandemics (Mheidly and Fares, 2020). Research on the factors contributing to media content’s effects on audience responses to the pandemic may be useful in public health education and the management of future pandemics. Therefore, this study examines the association between media content and fear of COVID-19 by addressing two central factors in research on the effects of news content: audience-protagonist similarity and social distance.

Audience-protagonist similarity refers to objective similarities between the audience member and the protagonist in characteristics such as demographic and background attributes (e.g., Fassnacht and Scharwey, 2015), whereas psychological distance refers to the audience member’s subjective perception of psychological closeness to or distance from the protagonist (Trope et al., 2007; Trope and Liberman, 2010; Razpurker-Apfeld and Tal-Or, 2026). In the present study, these constructs are therefore treated as conceptually and operationally distinct: audience-protagonist similarity is experimentally manipulated through the protagonist’s background characteristics, whereas perceived social distance is assessed following exposure to the news story as the participant’s subjective perception of psychological distance from the protagonist. Thus, the current study investigates whether experimentally manipulated audience-protagonist similarity is associated with fear of COVID-19 through its effect on perceived social distance.

### Fear of COVID-19

The concept of fear of COVID-19 refers to people’s concerns and anxieties about contact with individuals infected by COVID-19 (Lin, 2020), the possibility of contracting the virus, the possibility that family members contract this disease (Ahorsu et al., 2020; Lin, 2020; Liu et al., 2020; Presti et al., 2020; Shapiro et al., 2020), fears of death due to the virus (Horesh and Brown, 2020), and adverse economic outcomes due to pandemic-related lockdowns (Shapiro et al., 2020; Levy and Cohen-Louck, 2021). Research indicated that the fear of COVID-19 is positively associated with the virus’s transmission and mortality rates (Ahorsu et al., 2020). People directly impacted by the pandemic or those at risk of being personally impacted were more afraid of COVID-19 (Fitzpatrick et al., 2020; Sasaki et al., 2020). The fear of COVID-19 is associated with high emotional distress, depression, anxiety, and low life satisfaction and individual functioning (Presti et al., 2020; Shapiro et al., 2020; Wang et al., 2020; Braun-Lewensohn et al., 2021; Levy and Cohen-Louck, 2021; Levy, 2022; Cohen-Louck and Levy, 2023). Additionally, although high fear of COVID-19 was associated with avoidance of healthcare services (Muller, 2012; Harper et al., 2021), it was also associated with greater adherence to infection prevention and control strategies (Heiat et al., 2021). Thus, identifying the factors affecting fear of COVID-19 may facilitate more effective individual management of COVID-19-related fears and promote quality of life and mental health. Furthermore, a better understanding of the fear of COVID-19 can be used in health education to control and prevent viral pandemics by promoting public acceptance of preventive strategies and measures.

### Audience-protagonist similarity and social distance

During mass disasters, media outlets play a significant role in broadcasting fear and creating panic (Horesh and Brown, 2020; Cohen-Louck and Levy, 2021), and the consumption of disaster media increases adverse reactions such as depression, stress, anger, and acute grief (Pfefferbaum et al., 2014; Wormwood et al., 2019). The audience’s emotional distress may be generated by identification with the direct victims of mass disasters and their suffering since people often identify with protagonists in media reports, TV shows, movies, etc (Mares and Braun, 2013; Martins et al., 2020; e.g., Abdulaev and Shomron, 2022). Nevertheless, exposure to media does not necessarily lead to identification and depends on the audience’s interpretation of media content (Hall, 2003; Katyal, 2006). According to McGuire’s (1984) communication/persuasion framework, the processes through which public communication influences health-related responses and behavior depend on characteristics of the source, message, channel, receiver, and destination, which are considered important determinants of communication effectiveness (Kreuter and McClure, 2004). The present study focuses specifically on the relationship between receiver and message characteristics by examining audience-protagonist similarity and perceived social distance, and on whether social distance mediates the association between similarity and fear of COVID-19.

Audience-protagonist similarity is assumed to increase identification with the protagonist (De Graaf, 2014; e.g., Cohen et al., 2018; Razpurker-Apfeld and Tal-Or, 2024), particularly when the shared characteristics are meaningful to the audience member’s sense of identity or are relevant to the narrative context (Cohen et al., 2018). Such similarity may be based on demographic traits such as age, gender, race, ethnicity, etc (Thompson, 2003; Stuckelberger and Koedam, 2022), as well as physical appearance, name, personality traits, beliefs, and opinions (Eyal and Rubin, 2003; Hoffner and Buchanan, 2005; Williams, 2010; Howard and Kerin, 2011; e.g., Chen et al., 2016). The relevance of audience-protagonist similarity is also reflected in the broader literature on health communication. Narratives and personalized health messages may facilitate information processing, reduce resistance, and enhance the personal relevance of health information (Kreuter et al., 2007; Hawkins et al., 2008). Research has also examined similarity between recipients and message sources in health communication, although its effects may depend on message characteristics and context (Lu, 2013; Christy et al., 2022).

Importantly, audience-protagonist similarity may influence not only identification but also perceptions of health-related risk and severity. For example, participants’ perceptions of the risk of developing cancer varied based on the similarities between them and a cancer patient’s living arrangements. In the case of a patient living in similar living arrangements, participants perceived themselves to be more at risk of developing cancer than in the case of a patient living in dissimilar living arrangements (De Graaf, 2014). Likewise, in the case of caffeine overdose, similarity in age positively impacted identification, influencing severity perception (Chen et al., 2016). Accordingly, the following hypothesis was proposed:

H_1_: Participants exposed to news content featuring a protagonist with a similar background will report greater fear of COVID-19 than participants exposed to news content featuring a protagonist with a dissimilar background.

However, sometimes, the similarity in demographics between the audience and protagonist is not enough. For example, Cohen et al. (2018) examined the effect of audience-protagonist similarity on attitudes toward government responsibility for solving problems concerning the cost of living. This study’s findings showed that manipulating audience-protagonist similarity by combining two background details (e.g., gender and nationality) did not affect identification with the protagonist and attitudes (Cohen et al., 2018). Moreover, even when the effect of audience-protagonist similarity is found, it appears complex and sometimes conditioned on additional variables. For instance, Chen et al.'s (2016) findings about caffeine overdose suggest that age-based similarity had a more substantial effect on identification when combined with gender. Young participants were more likely to identify with a young protagonist of the same gender compared to an older protagonist of the opposite gender. Another example of the complex effect of audience-protagonist similarity shows that the degree of identification that video game players feel with their avatars is influenced not only by the visual resemblance between the avatar and the player but also by whether the player created the avatar themselves (Williams, 2010).

Thus, the association between audience-protagonist similarity and fear of COVID-19 may be more complex than a direct effect of objective similarity alone and may also depend on how similar or close participants perceive the protagonist to be. The present study therefore examines perceived social distance as a potential mechanism linking audience-protagonist similarity with fear. Construal Level Theory (CLT), which addresses the role of psychological distance in shaping individuals’ perceptions and responses, provides a particularly relevant theoretical framework for examining this mechanism.

According to CLT, psychological distance constitutes a foundational cognitive-perceptual mechanism that influences how individuals relate to events and others (Trope and Liberman, 2010). CLT proposes that psychologically close objects and events are represented more concretely, whereas psychologically distant objects and events are represented more abstractly (Trope et al., 2007; Trope and Liberman, 2010, 2012). At the concrete level, thinking is more detailed and contextualized, while at the abstract level, thinking involves more integrated and out-of-context details. The levels of thinking (concrete vs. abstract) are associated with different psychological distances (Trope et al., 2007; Trope and Liberman, 2010).

Psychological distance refers to the perceived degree of detachment from a phenomenon and comprises four dimensions: temporal, spatial, hypothetical, and social distance (Trope and Liberman, 2010, 2012). Temporal distance concerns how close or distant an event is perceived in time, while spatial distance refers to its physical or geographical proximity. Hypothetical distance refers to the perceived likelihood or possibility of experiencing circumstances similar to those faced by the people involved (Soderberg et al., 2015). The present study focuses specifically on social distance, which concerns perceived social and personal similarities between oneself and those involved (e.g., Spence et al., 2012).

Research examining psychological distance as a multidimensional construct has linked lower perceived psychological distance to stronger risk perceptions, worries, and fears. Specifically, psychological distance has been negatively associated with climate risk perception (Aslam and Rana, 2022), fear of climate change (Chu and Yang, 2019), worries about crime (Mellberg et al., 2022) and terrorism (Cohen-Louck et al., 2026), and fears of traveling during and following the COVID-19 pandemic (Li et al., 2020). Because these studies generally assessed psychological distance across multiple dimensions, however, they do not allow the specific contribution of social distance to be isolated.

Research focusing specifically on social distance nevertheless indicates that this dimension is associated with individual attitudes and behaviors (Ahmed, 2007; Trope et al., 2007; Liviatan et al., 2008; Fassnacht and Scharwey, 2015). Thus, for example, reduced social distance facilitates better salesperson-customer relationships and increases sales efficiency (Fassnacht and Scharwey, 2015). Additionally, social distance is related to participant perceptions of the protagonist’s behavior (Liviatan et al., 2008), the perceived relevancy of the protagonist’s recommendations for the near future (Zhao and Xie, 2011), and the tendency for discrimination toward a socially distant protagonist (Ahmed, 2007). Taken together, these findings suggest that perceived social distance may shape emotional responses to information concerning others and may therefore also be relevant to fear responses. Accordingly, we hypothesized the following:

*H_2_*: A negative association exists between social distance from a protagonist and fear of COVID-19: Greater social distance from a protagonist will be associated with lower levels of fear and vice versa.

Research suggests that social distance can be influenced through audience-protagonist similarity (Trope et al., 2007). Social distance can be reduced by creating similar demographic traits such as social status, income, or educational background (e.g., Fassnacht and Scharwey, 2015), but it is not the only way to reduce social distance. For instance, participants perceived a student attending the same courses (similar protagonist) as significantly closer to themselves than a student attending different courses (a dissimilar protagonist; Liviatan et al., 2008). Additionally, social distance can be reduced through affiliation with a protagonist’s social group (Ahmed, 2007; Trope et al., 2007; Zhao and Xie, 2011). Considering these findings, it is possible to assume that perceived social distance from a protagonist in a news story about the effect of COVID-19 can be affected by audience-protagonist similarity in demographic characteristics such as gender, age, country of residence, and health background. Therefore, this study’s hypothesis is as follows:

*H_3_*: Participants exposed to news content featuring a protagonist with a similar background will report lower perceived social distance from the protagonist than participants exposed to news content featuring a protagonist with a dissimilar background.

Furthermore, considering that audience-protagonist similarity is negatively related to social distance (Trope et al., 2007; Liviatan et al., 2008; e.g., Fassnacht and Scharwey, 2015) and that psychological distance, including social distance, affects fears and risk perception (Chu and Yang, 2019; e.g., Aslam and Rana, 2022; Mellberg et al., 2022), it is possible to assume that social distance mediates the relationship between audience-protagonist similarity and fear of COVID-19. Therefore, this study hypothesizes the following:

*H_4_*: Social distance will mediate the association between audience-protagonist similarity in COVID-19 news content and fear of COVID-19, such that exposure to a similar protagonist will be associated with lower perceived social distance, which in turn will be associated with greater fear of COVID-19 (Figure 1).

**Figure 1:**
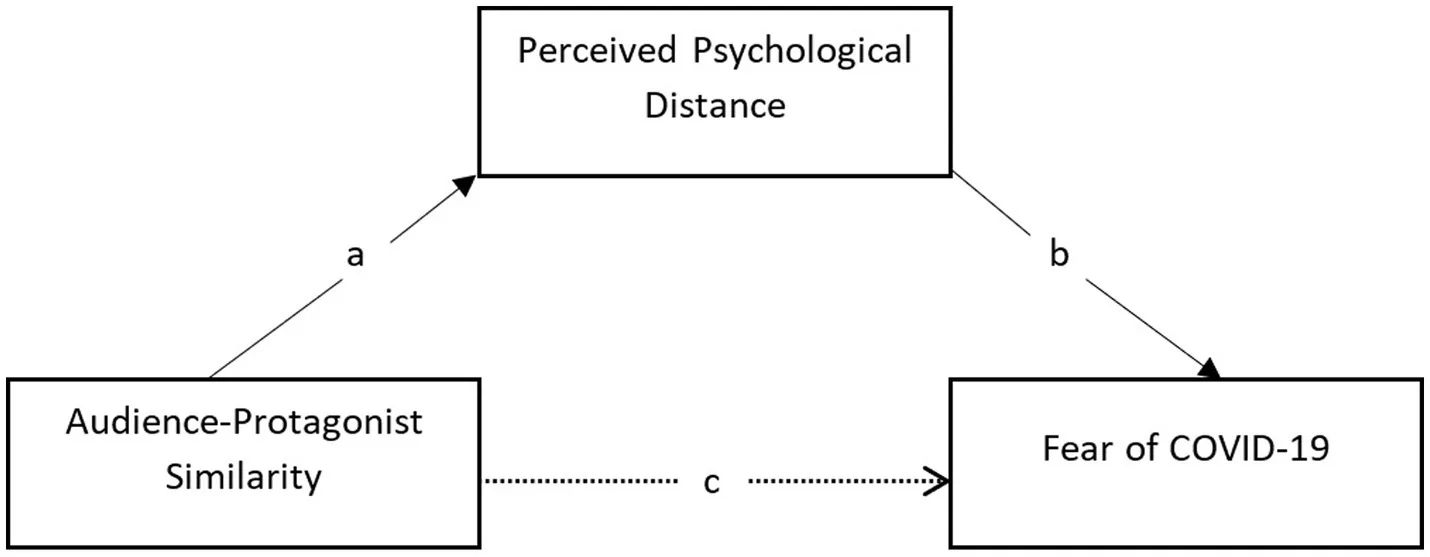
The model for prediction of fear of COVID-19- the mediating role of psychological distance.

### The current research

This study examines the interrelationships among audience-protagonist similarity, social distance from the protagonist, and fear of COVID-19. It extends prior research in several ways. First, although psychological distance has been linked to fear and risk perception, the role of audience-protagonist similarity in shaping fear responses to health-related news content remains understudied. The present study therefore examines whether exposure to a similar rather than dissimilar protagonist is associated with greater fear of COVID-19. Second, while some studies have empirically examined the relationship between similarity and social distance (e.g., Liu and Yang, 2023), others have treated similarity as an indicator of social distance rather than examining the two constructs separately (e.g., Ahmed, 2007; Fassnacht and Scharwey, 2015). The current study therefore distinguishes between experimentally manipulated audience-protagonist similarity and participants’ subsequent subjective perceptions of social distance.

A further contribution concerns the similarity manipulation itself. Previous studies have typically manipulated one or two characteristics (De Graaf, 2014; e.g., Chen et al., 2016; Cohen et al., 2018), whereas the present study combines multiple characteristics. Five participant characteristics were used to match the vignette to the respondent, including gender, age, smoking status, overweight status, and underlying health conditions, while country of residence was determined by experimental condition. This design enables a stronger contrast between similar and dissimilar protagonists and enables us to examine social distance as a distinct psychological response. Finally, the study tests whether perceived social distance mediates the association between audience-protagonist similarity and fear of COVID-19. In doing so, the study examines not only whether similarity is associated with fear, but also whether this association may operate through participants’ subjective perception of social closeness to the protagonist. These relationships may have potential implications for the design of health-related news content and preventive communication.

## Method

### Participants

This study included 84 respondents. The age range was 18–30 (*Mean* = 25.73, *SD* = 3.03), and half of the participants were female. Most participants were married or living with a spouse (67%), 31% were single, and 2% were divorced. Regarding religiosity, most participants were secular (61%), 21% traditional, and 18% religious. Approximately a quarter of the participants (26%) reported a high school level of education, 12% reported a tertiary level, approximately half (49%) had a B.A., and 13% had an M.A.

### Measures

#### Demographic characteristics

This part of the questionnaire gathered information about gender, age, marital status, level of religiosity, and education.

#### Health background

To assess the participants’ background concerning health conditions that could increase the risk of death due to COVID-19, they were asked to indicate whether they smoke and if they have any of the following health conditions: diabetes, heart disease, high blood pressure, immune system-related diseases, and obesity.

#### Scenarios

To examine the effect of audience-protagonist similarity, we created a vignette (Appendix A) that presented a news story about a protagonist who experienced symptoms of COVID-19 (dizziness, cough, and fever) and was taken by a spouse for a COVID-19 test. The news story stated that the test result was positive; therefore, this individual was admitted to a hospital. Later, the protagonist developed respiratory distress and was treated through mechanical ventilation. After a three-month hospitalization, this individual was released home for further treatment and respiratory rehabilitation at the community clinic. Experimental vignette methodology enables researchers to systematically manipulate theoretically relevant characteristics within realistic scenarios while maintaining experimental control over the stimulus (Aguinis and Bradley, 2014), consistent with the use of experimental designs to examine responses to media content in media psychology research (Prot and Anderson, 2013).

#### Audience-protagonist similarity manipulation

To manipulate the degree of similarity between the participant and the protagonist, we varied the protagonist’s gender (female/male), age (25/40), overweight status, presence or absence of underlying health conditions, smoking (smoking/no smoking), and country of residence (Israel/Germany). The first five characteristics (gender, age, overweight status, health conditions, and smoking) were matched to participants’ questionnaire responses, whereas country of residence was determined by experimental condition: Israel in the similarity condition and Germany in the dissimilarity condition. Thus, the manipulation combined similarity in personal and social-background characteristics with similarity in national-geographical context. In the similarity condition, the protagonist’s demographic and health characteristics were matched as closely as possible to those reported by the participant, whereas in the dissimilarity condition, these characteristics were selected to maximize dissimilarity. Because all participants were Israeli, country of residence was also used to distinguish the two conditions, with an Israeli protagonist presented in the similarity condition and a German protagonist in the dissimilarity condition. Country of residence formed part of the objective audience-protagonist similarity manipulation and was not used as a measure of participants’ perceived spatial psychological distance. The detailed matching and assignment procedure is described in the Procedure section.

Gender was manipulated using gender-associated names (Dafna and Eva for the female version; Shlomo and Hans for the male version). Country of residence was manipulated through the news website’s name (YNET for the Israeli version vs. Der Spiegel for the German version), the protagonist’s first and last names (Dafna/Shlomo Abrahamov vs. Eva/Hans Fritz), and the stated place of residence (Petah-Tikva vs. Hamburg, Germany). Germany was selected to provide a clearly foreign European context that would be readily distinguishable from the participants’ Israeli context, while remaining sufficiently familiar and plausible as the setting of a COVID-19 news report. The choice was not based on any Germany-specific theoretical assumption.

#### Attention and comprehension check

To ensure that the participants read the vignette and understood it, we included three questions regarding the story’s details: (1) What was the occupation of the individual in the story, (2) Which physiological symptoms did the individual experience before the COVID-19 test, and (3) How long was the individual hospitalized? Participants who did not answer these questions correctly were excluded from the analyses.

#### Social distance

The present study assessed the social distance from the protagonist. Participants rated both their perceived resemblance to the protagonist (“To what degree did you feel that the person in the article resembles you?”) and their perceived closeness to the protagonist (“To what degree did you feel close to the person in the article?”). Both items were treated as indicators of perceived social distance. Responses to both items were provided on a Likert scale from 1 (very low degree) to 5 (very high degree). The correlation between similarity and closeness ratings was strong (*r* = 0.75, *p* < 0.001). We recoded the two items so a higher score reflected perceiving the protagonist as more socially distant, and then calculated a combined perceived social distance score by averaging the two items.

#### Fear of COVID-19

Fear of COVID-19 (FCV-19CS; Ahorsu et al., 2020) is a self-report measure that assesses fear of COVID-19. This scale included seven items referring to the emotional aspects of fear responses toward the pandemic. The participants’ responses ranged from 1 (strongly disagree) to 5 (strongly agree). The Hebrew version of the scale was validated for use in the Israeli population (Tzur Bitan et al., 2020). In the current study, Cronbach’s alpha was 0.92.

### Procedure

#### Data collection

The online survey for this study was conducted between 2 and 3 January 2022. By this time, the Israeli public went through three general lockdowns (March–April 2020, September–October 2020; December–January 2021) and three waves of vaccination (Vinrab, 2022). Additionally, January 2022 was marked by the spread of Delta and Omicron (David J. Sencer CDC Museum, 2023) and a relatively high number of new COVID-19 patients (Vinrab, 2022). To ensure that the participants paid attention to the details of the vignette, we examined participants’ answers to the attention and comprehension check questions. Out of the initial 84 participants, six were excluded from the subsequent analyses based on the predefined attention and comprehension criteria, yielding a final analytic sample of 78 participants. Participants were recruited through GeoCartography’s registered online respondent panel. According to the panel provider, technical safeguards were in place to prevent duplicate participation and bot-generated responses.

#### Ethics

The Ariel University Ethics Committee approved this study (AU-SOC-KL-20210321). At the beginning of the survey, the questionnaires stated that participation in this study was anonymous, the responses were confidential, and the data would serve only research purposes. All respondents gave their informed written consent to participate in this study. The survey company (GeoCartography Knowledge Group) compensated the participants for their participation in this study as part of their ongoing participation in the online panel.

#### Survey flow and research manipulation

Each participant was exposed to one vignette only and was randomly assigned to one of two experimental conditions: a similar-protagonist condition or a dissimilar-protagonist condition. The background characteristics used for vignette matching were collected before presentation of the vignette. After reading the vignette, participants were required to wait 30 s before proceeding and could not return to the vignette once they advanced. They then completed the attention and comprehension check, followed by the perceived social-distance measure and the Fear of COVID-19 Scale (FCV-19CS).

Since all our participants were Israeli, the similar condition featured an Israeli protagonist whose demographic and health characteristics were matched as closely as possible to those reported by the participant. The dissimilar condition featured a German COVID-19 patient whose demographic and health characteristics were selected to maximize dissimilarity from those of the participant.

For example, if a female participant reported that she did not smoke and did not have any health issues that increase the risk of COVID-19-related death, then in a *similar* condition, she was presented with a story about an Israeli woman (Dafna) who did not smoke and did not have any prior health issues. However, if a participant were presented with a dissimilar vignette, the patient would be described as a German male (Hans) who smoked and had health issues before COVID-19. All possible vignette combinations (64 versions) were created in advance. These 64 versions represented pre-created combinations of protagonist characteristics used to implement the two experimental conditions (similar and dissimilar) and did not constitute 64 separate experimental groups or analytic cells. For example, a 25-year-old female participant who did not smoke and had no underlying health conditions could receive, in the dissimilar condition, a vignette describing a 40-year-old German male who smoked and had underlying health conditions. Conversely, a 40-year-old male participant who smoked and had an underlying health condition could receive, in the same dissimilar condition, a vignette describing a 25-year-old German female who did not smoke and had no underlying health conditions. Thus, different vignette versions could represent the same experimental condition because similarity or dissimilarity was defined relative to each participant’s characteristics. Participants were first randomly assigned to either the similarity or dissimilarity condition. The survey then used each participant’s reported gender, age group, smoking status, overweight status, and presence or absence of underlying health conditions to select, from the pre-created versions, the vignette that best matched the assigned condition by maximizing either similarity or dissimilarity between the participant and the protagonist. Country of residence was determined by experimental condition, with an Israeli protagonist in the similarity condition and a German protagonist in the dissimilarity condition. The five participant-based matching characteristics were considered jointly rather than according to a predefined hierarchy of priority (see Appendix B).

#### Statistical analyses

Statistical analyses were conducted using SPSS. Group differences were examined using independent-samples *t* tests. The hypothesized indirect effect of audience-protagonist similarity on fear of COVID-19 through perceived social distance was tested using PROCESS Model 4 with 5,000 bootstrap resamples. A sensitivity power analysis based on the final sample of 78 participants (44 in the dissimilar condition and 34 in the similar condition) indicated that, assuming a two-sided α = 0.05 and 80% power, the study could detect between-group effects of approximately Cohen’s d = 0.65 or larger. Statistical assumptions were examined prior to interpretation of the inferential analyses. Because the social-distance scores showed unequal variances across conditions, between-group differences in social distance were evaluated using Welch’s t test. Regression diagnostics indicated no evidence of heteroscedasticity. Given departures from normality, robustness analyses were also conducted using HC3 heteroscedasticity-robust standard errors; these yielded the same pattern of statistical conclusions as the primary analyses.

## Results

### Data quality test and assumption checks

First, we examined whether the participants were attentive to the information in the vignette. Most participants (86%) responded correctly to the three multiple-choice questions about the vignette. Participants in the two conditions of the different vignettes did not differ in their understanding of the details concerning the question about the protagonist’s profession) $\chi_{\left(2\right)}^{2}$ =0.55, *p* = 0.760(, the question about the protagonist’s physiological symptoms ( $\chi_{\left(1\right)}^{2}$ =0.03, *p* = 0.864), and the question about the period of hospitalization ( $\chi_{\left(1\right)}^{2}$ =0.06, *p* = 0.806). Answering these questions correctly (or not) did not depend upon the vignette’s condition ( $\chi_{\left(1\right)}^{2}$ =0.09, *p* = 0.761).

Prior to the inferential analyses, statistical assumptions were examined. The study variables showed departures from normality, and social-distance scores showed unequal variances across conditions. Accordingly, the between-group comparison of social distance was conducted using Welch’s t test. Nonparametric sensitivity analyses of the between-group comparisons yielded the same substantive conclusions. Regression diagnostics indicated no evidence of heteroscedasticity, and robustness analyses using HC3 heteroscedasticity-robust standard errors yielded the same pattern of statistical conclusions as the primary analyses. One univariate outlier was identified; excluding this case did not alter the substantive conclusions.

### Hypothesis testing

We first examined whether audience-protagonist similarity had a direct effect on fear of COVID-19 (H1). Contrary to H_1_, an independent-samples *t* test showed no significant difference in fear of COVID-19 between the similar- and dissimilar-protagonist conditions, *t*(76) = 0.597, *p* = 0.552. Next, we examined the association between perceived social distance and fear of COVID-19 (H2). Consistent with H_2_, perceived social distance was negatively correlated with fear of COVID-19, *r*(76) = −0.30, *p* = 0.007, 95% CI [−0.49, −0.09]. A Spearman rank-order correlation yielded the same substantive conclusion, *ρ* = −0.28, *p* = 0.012. We then examined whether the two vignette conditions generated the hypothesized difference in perceived social distance (H_3_). As hypothesized, a Welch independent-samples *t-test* showed a significant difference between conditions, *t*(54.56) = 2.922, *p* = 0.003, Cohen’s *d* = 0.70. Participants exposed to a dissimilar protagonist (*n* = 44) reported greater perceived social distance (*Mean* = 4.34, *S.D.* = 0.81) than participants exposed to a similar protagonist (*n* = 34, *Mean* = 3.63, *S.D.* = 1.22).

Our central mediation hypothesis (H_4_) proposed that perceived social distance would mediate the association between audience-protagonist similarity and fear of COVID-19. Specifically, we expected exposure to a similar protagonist to be associated with lower perceived social distance, which in turn would be associated with greater fear of COVID-19. We tested this hypothesis using a mediation model with the SPSS PROCESS macro (Model 4 with 5,000 bootstrapped resamples; Hayes, 2022). Audience-protagonist similarity condition served as the independent variable, perceived social distance as the mediator, and fear of COVID-19 as the dependent variable. Exposure to a similar protagonist was associated with lower perceived social distance (*b* = −0.71, *S.E.* = 0.23, *p* = 0.003) and greater perceived social distance was associated with lower fear of COVID (*b* = −0.30, *S.E.* = 0.09, *p* = 0.002). The total effect of similarity condition on fear was not significant (*b* = −0.12, *S.E.* = 0.20, *p* = 0.553, 95% *CI* [−0.515, 0.278]), nor was the direct effect (*b* = −0.33, *S.E.* = 0.20, *p* = 0.101, 95% *CI* [−0.728, 0.066]). However, the indirect effect through perceived social distance was significant (*I.E.* = 0.21, *S.E.* = 0.11, 95% *CI* [0.042, 0.453]), supporting H_4_ (see Figure 2). Thus, exposure to a similar rather than dissimilar protagonist was associated with greater fear of COVID-19 indirectly through lower perceived social distance from the protagonist.

**Figure 2:**
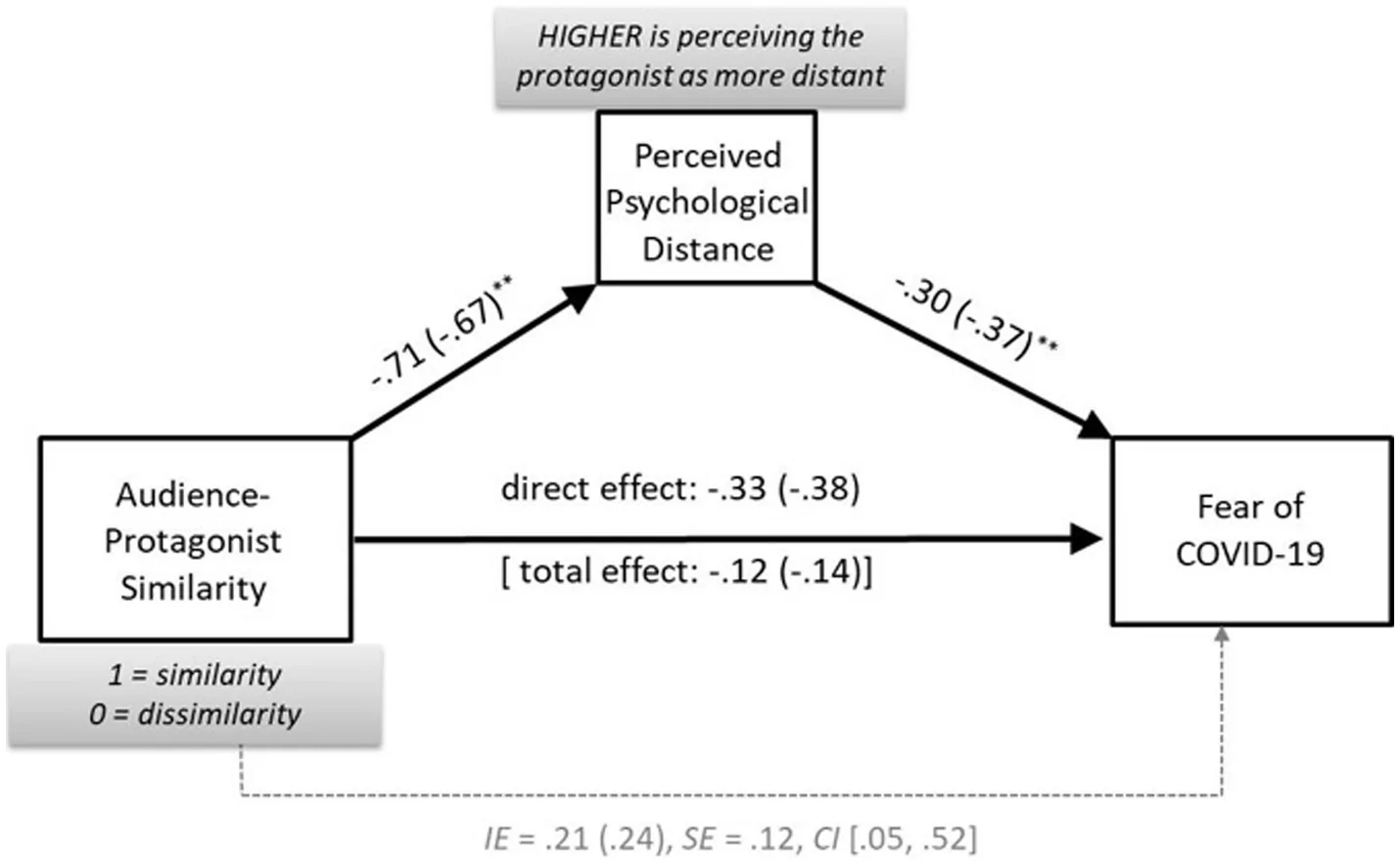
A mediation model demonstrating the effect of audience-protagonist similarity on fear of COVID-19 through perceived psychological distance. A mediation model (Process Model 4) was conducted to test the main hypothesis (*N* = 78). Unstandardized regression coefficients are presented with standardized coefficients (in brackets). The direct and total effects (in square brackets) were nonsignificant, while the indirect effect (gray dotted line) was significant. CI represents 95% confidence intervals. **p <0.01.

## Discussion

This study examined the interrelationship between audience-protagonist similarity, social distance, and fear of COVID-19. Our findings demonstrate the significance of individual perceptions regarding protagonists in news and informative content, particularly in medical contexts. If the goal is to enhance awareness of medical situations such as the COVID-19 pandemic and encourage the public to seek medical care and adopt preventive measures such as vaccination, it may be important to ensure that people can relate to protagonists and perceive them as close and similar. Policymakers and professionals in charge of promoting public compliance with health-related preventive measures may consider providing medical infomercials and informative stories, allowing people from target populations to find commonalities with protagonists.

Our findings support H_3_ and show that audience-protagonist similarity was associated with lower perceived social distance. This finding corresponds to prior findings showing that audience-protagonist similarity in the social background can decrease social distance (Trope et al., 2007; Liviatan et al., 2008; Zhao and Xie, 2011; Fassnacht and Scharwey, 2015). From a methodological point of view, our finding may shed some light on why similarity manipulation did not affect identification in Cohen et al.'s (2018) study. It may be that a meaningful engagement between the audience and the protagonist requires manipulation of audience-protagonist similarity through more than two background variables. Our findings indicated that a manipulation combining multiple participant-based characteristics together with country of residence (gender, age, health background, smoking status, and country of residence) produced a significant difference in social distance.

Consistent with H_2_, greater perceived social distance from the protagonist was associated with lower fear of COVID-19. This finding is consistent with previous research linking lower psychological distance with stronger risk perceptions, worries, and fears, and extends this pattern to perceived social distance in the context of COVID-19 news content. Nevertheless, similar to Cohen et al.'s (2018) findings that objective similarity did not affect attitudes, and contrary to our hypothesis (H_1_), the findings show that merely presenting information with protagonists who are objectively similar to the audience does not increase fear of COVID-19. It appears that the protagonist’s effect on audiences depends on audience perceptions of their similarity to the protagonist. Our findings on the mediating role of social distance (H_4_) demonstrate that perceived closeness to the protagonist plays an important role in linking informative text with fear of disease. This finding corresponds with Hall's (2003) argument that audiences are not passive and that the effects of media content depend on subjective perception and interpretation (Hall, 2003; Katyal, 2006), as well as CLT’s claim that psychological distance is central to the individual experience of events and phenomena (Trope and Liberman, 2010).

### Limitations and future research

This study is not without limitations. First, although the sample size was adequate for detecting medium-to-large effects, its relatively small size limited the study’s sensitivity to detect smaller effects (Lakens, 2022). Future studies with larger samples may also examine the robustness of these associations using alternative modeling frameworks, including Bayesian approaches. Secondly, our focus on fear of COVID-19 and the specific choice of similarity-inducing factors may limit the external validity of the findings in the context of health-related media messages. Future studies should explore our model in the context of other diseases or traumatic events (e.g., terrorism, crime) and examine whether there are additional significant variables that may increase audience-protagonist similarity and reduce audience-protagonist social distance. Thirdly, this study addressed only the social dimension of psychological distance (perceived similarity and closeness to the protagonist), and future studies should explore additional dimensions of psychological distance, including temporal, spatial, and hypothetical distances. Future studies should also identify the minimum number of similarity-inducing factors required and examine whether these factors vary in their relevance to specific health contexts. Additionally, cultural and social context may affect the significance of some similarity-inducing factors, and therefore, future studies should explore our model in additional cultural contexts.

## Conclusion

This study’s findings facilitate a better comprehension of the association between media messages and public fears of pandemics. Furthermore, our findings provide empirical evidence for the mediating role of social distance in the association between audience-protagonist similarity and fears of a pandemic. From a practical point of view, this study’s findings may be relevant for creating effective media messages that encourage people to seek medical care and adopt preventive measures such as vaccination, not only for COVID-19 but also for other diseases, such as influenza.

Beyond medical concerns, these findings have broader practical implications, specifically for designing effective communication strategies to inform the public about science-related topics, raise awareness of scientific issues, and develop targeted messages more likely to resonate with different audiences. Furthermore, understanding the association between similarity and social distance can also be relevant for creating messages that minimize public fear and anxiety in health-related contexts and across a wide variety of traumatic experiences.

## Data Availability

The raw data supporting the conclusions of this article will be made available by the authors, without undue reservation.
