# Supplementary material for: The mediating role of social distance on the association between audience-protagonist similarity and fear: a case of fear of COVID-19
Source: Front Psychol. 2026 Oct 14;17:1823941. doi: 10.3389/fpsyg.2026.1823941 (PMC13749118; doi:10.3389/fpsyg.2026.1823941)
Supplement: Supplementary file 1 [file Supplementary_file_1.DOCX]

**Appendix A**

Vignette

The following story is an extract from the news report published on YNET (Der Spiegel): "Shlomo (Dafna/Eva/Hans) Avraham (Fritz), a resident of Petah Tikva (Hamburg, Germany), 40 years old (25 years old), who has an illness (no background illness) and smokes (does not smoke), works in the cafeteria at the District Court in Tel Aviv/Hamburg: "About four months ago, I felt dizzy during the work day. At first, I did not attach any importance to it and thought it was related to the stress I had recently experienced and lack of sleep, but in the evening, when I returned home, I started coughing and felt that I had a fever. My wife (husband) drove me to the hospital, where they performed a test for COVID-19. The result came out positive and I was quite stressed. Although I felt relatively fine, the doctor decided to keep me under observation. The next morning, I started having difficulty breathing, and I was weak and could not eat. The breathing difficulties got worse in the afternoon, and I felt that I needed help breathing, so they hooked me up to a ventilator. After a three-month hospitalization, I was released home for further treatment and respiratory rehabilitation at the community clinic."
