# Supplementary material for: The mediating role of social distance on the association between audience-protagonist similarity and fear: a case of fear of COVID-19
Source: Front Psychol. 2026 Oct 14;17:1823941. doi: 10.3389/fpsyg.2026.1823941 (PMC13749118; doi:10.3389/fpsyg.2026.1823941)
Supplement: Supplementary file 2 [file Supplementary_file_2.DOCX]

Appendix B

Table 1b. ***Illustrative examples of vignette assignment in the similar and dissimilar conditions***

| Participant profile | Experimental condition | Protagonist profile presented |
| --- | --- | --- |
| Female, age 25, non-smoker, no overweight, no underlying health condition | Similar | Israeli female, age 25, non-smoker, no overweight, no underlying health condition |
| Female, age 25, non-smoker, no overweight, no underlying health condition | Dissimilar | German male, age 40, smoker, overweight, with an underlying health condition |
| Male, age 40, smoker, overweight, with an underlying health condition | Similar | Israeli male, age 40, smoker, overweight, with an underlying health condition |
| Male, age 40, smoker, overweight, with an underlying health condition | Dissimilar | German female, age 25, non-smoker, no overweight, no underlying health condition |

***Note*.** The examples illustrate the matching algorithm. Participants were first randomly assigned to the similar or dissimilar condition, after which a pre-created vignette was selected on the basis of gender, age group, smoking status, overweight status, and presence or absence of underlying health conditions. Country of residence was Israeli in the similar condition and German in the dissimilar condition.
